# A148 OSTEOSARCOMA GASTROINTESTINAL METASTASIS: A RARE ENTITY IN ADULTS

**DOI:** 10.1093/jcag/gwad061.148

**Published:** 2024-02-14

**Authors:** B Alabdulkarim, F Habal

**Affiliations:** Gastroentertology, University of Toronto, Toronto, ON, Canada; Gastroentertology, University of Toronto, Toronto, ON, Canada

## Abstract

**Background:**

Osteosarcoma is highly aggressive and is the most common primary bone malignancy for both children and adults with a bimodal age distribution. The first peak coincides with pubertal growth spurt and the second is in the seventh and eighth decade of life. They frequently metastasize to the lungs. Metastasis to the gastrointestinal tract is extremely rare and to our knowledge, there have only been 15 cases reported in the literature since 1987, of those only three in the second peak.

**Aims:**

Describe a case of 61-year-old female with periampullary metastatic osteosarcoma. Describe the demographics of osteosarcoma patients who develop gastrointestinal metastasis.

**Methods:**

Search Pubmed using the terms: ("Osteosarcoma"[MeSH Terms] OR "Osteosarcoma"[Title/Abstract]) AND ("Gastrointestinal Tract"[MeSH Terms] OR "Gastrointestinal Tract"[Title/Abstract] AND "Case Reports"[Publication Type]. 113 articles were identified and screened by title. 15 articles were further screened by abstract or full text. 7 articles were identified. the remainder 7 articles were located by screening the references of the previously identified articles.

**Results:**

A 61-year-old woman with a medical history significant for metastatic Osteosarcoma was referred to Gastroenterology clinic July 2023 for anemia investigation. Her Osteosarcoma was first diagnosed in the left femur in 2009. She was known for extensive bilateral lung metastasis, subcutaneous metastatic deposits, and distant bone metastasis. She had undergone over 37 surgeries and multiple sessions of radiotherapy. Aside from neoadjuvant and adjuvant chemotherapy for the primary tumor and the first occurrence of lung metastasis, she refused to undergo further cycles of chemotherapy. She had a 60 mg/dl hemoglobin drop from a previous one in December 2023 and intermittent melena. On gastroscopy, she was found to have a 2 cm bleeding mass in the periampullary area. The pathology report of the biopsies was consistent with metastatic osteosarcoma. It showed fragments of ampullary mucosa incorporating a malignant spindle cell neoplasm with a fascicular growth pattern, including scattered osteoclast-like giant cells, morphologically consistent with the known history of metastatic osteosarcoma.

Of the 15 cases published, 9 were in patients 27 years old and younger, 3 were in patients 50 and older, and 3 were only available as titles. 50% of cases presented with intussusception while the rest had anemia or melena. The site of metastasis was the jejunum in the majority of patients (58%). Other sites included the ileum, stomach, colon, and esophagus. Only one other patient had periampullary metastasis. All patients had lung metastasis.

**Conclusions:**

Gastrointestinal metastasis of osteosarcoma remains a rare occurrence. Our case report describes the second case reported in the literature of periampullary metastasis and the first case reported in older patients

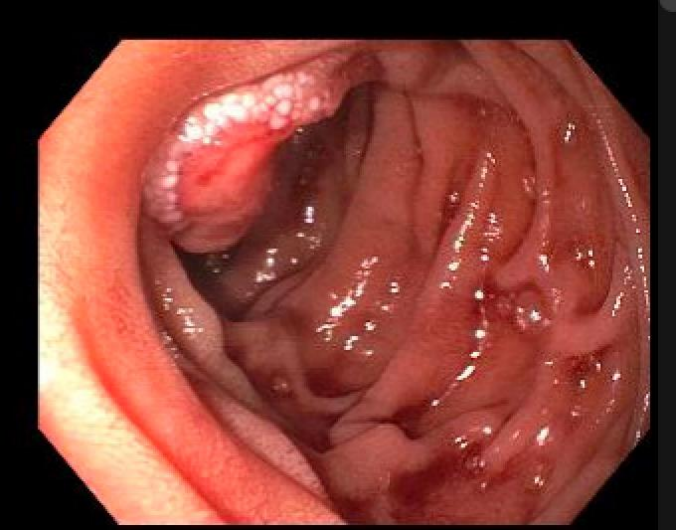

**Funding Agencies:**

None

